# Healthcare workers’ perspectives on diabetic foot complications among type 2 diabetes mellitus patients in Fiji

**DOI:** 10.1371/journal.pone.0307972

**Published:** 2024-09-23

**Authors:** Suliana Saverio, Masoud Mohammadnezhad, Filimone Raikanikoda

**Affiliations:** 1 Sigatoka Hospital, Fiji Ministry of Health and Medical Services, Sigatoka, Fiji; 2 School of Nursing and Midwifery, Birmingham City University, Birmingham, United Kingdom; 3 Department of Health Education and Behavioral Sciences, Faculty of Public Health, Mahidol University, Nakhon Pathom, Thailand; 4 Department of Public Health, Daffodil International University (DIU), Daffodil Smart City (DSC), Birulia, Savar, Dhaka, Bangladesh; 5 School of Public Health and Primary Care, Fiji National University, Suva, Fiji; University of Education Winneba Faculty of Science Education, GHANA

## Abstract

**Introduction:**

Diabetic Foot Complications (DFCs) are a growing cause of morbidity and mortality with less than one third of physicians able to discern the signs of diabetes related peripheral neuropathy. DFCs and resultant amputations account for a considerable proportion of surgeries in Fiji, with very limited literature available to verify the factors that influence these alarming figures. This study aimed to explore Health Care Workers’ (HCWs) perspectives on diabetic foot complications and challenges of foot care management in Fiji.

**Method:**

An exploratory descriptive qualitative design was used among HCWs at the Sigatoka Sub Divisional Hospital (SDH), Fiji in 2021. HCWs at the SDH were required to have a minimum work experience of at least six months in public health. All participants who met the inclusion criteria were selected through purposive sampling. Data was collected using a focus group discussion guide composed of semi-structured open-ended questions to guide the Focus Group Discussions (FGDs). Focus discussions were audio recorded and transcribed with thematic analysis applied to derive the themes and sub-themes outlined in the study.

**Results:**

Twenty HCWs participated in four FGDs with four major themes identified. The first theme was HCWs’ perceptions and practice of foot care which revealed that all participants had adequate diabetic foot care knowledge. The second theme was factors affecting foot care which was mainly focused on identified barriers such as inadequate patient foot care knowledge, the lack of resources such as manpower, and health system challenges like the COVID-19 pandemic. The third theme is creating awareness among patients and HCWs to improve foot care practices. The fourth theme is strengthening foot care practices at the different levels of health care that is aimed at optimizing diabetic foot outcomes.

**Conclusion:**

Various foot care barriers namely patient factors and the lack of resources is a concern depicted in this study. There is a need to address health system barriers and enforce diabetic foot education, screening and care for patients and the community.

## Introduction

Diabetes Mellitus (DM) is a major health concern which is increasing worldwide– 420 million diagnosed diabetics today with projections of 570 million diabetics in 2030, and a further increase to 700 million by 2045. DM is the 9^th^ leading cause of death worldwide and has increased by 70% since 2000 [1, 2]. Diabetic Foot Complications (DFCs) are an increasing cause of morbidity and mortality worldwide affecting 40 to 60 million people [3]. The global prevalence of DFCs ranges from 3% in the Oceania to as high as 13% in North America, with a global average of 6.4% [3, 4]. However, less than one third of physicians are able to discern the signs of diabetes related peripheral neuropathy so delayed or missed diagnosis contributes to the high morbidity and mortality rates of DFCs [3]. Few Healthcare Workers (HCWs) are aware of the necessity to routinely check patients’ feet and promote basic foot care practices [3, 5]; and routine foot examinations are a lowly perceived need unless a lesion is present, with low risk patients receive little to no information on foot care during consultations [6, 7]. This brews distrust in HCWs and the health system because ill-informed patients only learn of the seriousness of diabetes and the dangers of foot complications when they develop Diabetic Foot Ulcers (DFUs) [6]. Deguchi, et al., (2021) also found that poor patient-doctor communication resulted in low level of satisfaction with treatment [8]. Refresher courses can help boost HCWs’ confidence in conducting foot screening and patient foot care education [9–11].

HCWs play such an important role in promoting diabetes self-care and foot care practices to patients, their families and the community as a whole; so diabetes and foot care advice should be delivered in a language that improves a patient’s health literacy and is culturally appropriate and feasible for patients and their families to adhere to [10]. Many HCWs fail to establish good rapport and lack holistic care because appointments were often rushed during busy clinics [12] and care was mainly focused on medications alone without consideration or support for the emotional challenges patients face when dealing with diabetes and its complications [11]. With multidisciplinary approaches, medical advice from different disciplines confuses patients and untimely appointments serve as additional costs to patients and their families–which is one of the reasons patients abandon treatment [10].

It was widely reported that HCWs often overlooked examining patients’ feet and they neglected patient education on foot care or failed to reinforce patient knowledge; so patients considered foot care as a low priority and failed to practice it [10, 11]. To help patients better understand foot self-care practices, demonstrations and visualizations of foot complications can be offered as a way of empowering them; and a running commentary during a comprehensive foot examination can be conducted so patients are reassured and aware of what foot care encompasses [6]. This is in line with recommendations for structured foot care education which is aimed at improving patient foot care knowledge and self-care behaviour [13]. Patients should also be given ample time to ask questions and clarify any remaining doubts [6] regardless of the duration of diabetes [14]. Establishing good rapport with patients is equally important as a trust-worthy doctor-patient relationship can encourage treatment adherence, improve patient satisfaction, and continuity of care [15–19].

The significance of DFCs and amputations in Fiji despite ongoing public health action is a concern that is reflected in the fact that 40% of all surgeries in Fiji are due to diabetic amputations, with one amputation occurring every 12 hours [20, 21]. This may be attributed to poor education, awareness, and foot care practices among patients and HCWs alike. The cost of care of DFUs is considerable because treatment costs span the duration of admission or outpatient wound care until rehabilitation; and includes traveling costs for hospital follow-ups, medications and dressing materials [11]. The recent impact of COVID-19 on the nation has left many more families struggling to make ends meet, let alone shoulder the financial burden of DFUs and lower limb amputations. The cost of care for DFUs creates a vicious cycle that places more financial strain on patients and their families; and generates social and psychological stress in the household [22]. Patient care suffers due to financial constraints, and inadequate wound care and follow-up and they are at risk of worse outcomes like severe infection leading to amputations. This underpins how important a role HCWs play in the management of DFCs, which needs to transition from a curative to preventative approach through early identification and referral of the high-risk diabetic feet through routine comprehensive diabetic foot examinations. Factors influencing DFCs and resultant amputations are common knowledge amongst HCWs in Fiji, with very limited literature to verify these facts. This study was conducted at a semi-urban health facility (Sigatoka Sub Divisional Hospital) on the main island of Viti Levu. The information yielded here can be compared to a similar study done recently at a rural health setting in Fiji [23]. This study explored HCWs’ perspectives on DFCs and challenges of foot care management in Sigatoka, Fiji. The findings of this study provide baseline information which may be used to guide future actions towards alleviating the morbidity and mortality of DFCs in Sigatoka; and may assist in creating policies that promote and support foot care.

## Methodology

### Study design and setting

An exploratory descriptive qualitative design using Focus Group Discussions (FGDs) was conducted to explore HCWs’ perceptions of DFCs, and foot care knowledge and practices from 3^rd^ September to 30^th^ September in 2021. FGDs were a more efficient and effective tool for data collection given the time constraint, but more so for the information yielded through group interaction that explored participants’ perceptions of foot care among colleagues [24, 25]. Power imbalance amongst participants were taken into consideration so participants were grouped accordingly to allow open and free dialogue. Validation from other colleagues through FGDs can encourage them to express their opinions freely on the reality of diabetic foot care in Sigatoka. The study was conducted at the Sigatoka Sub Divisional Hospital (SDH), which is the referral center for thirteen peripheral health facilities in the Nadroga-Navosa medical area. Sigatoka is one of five towns in the western division that caters to a population of 58,931 people [26]. The Consolidated Criteria for Reporting Qualitative Research (COREQ) was applied to ensure rigor of this study [27].

### Study sample

The SDH has 13 doctors (six males and seven females) and 55 nurses (4 males and 51 females) all of whom have attended to Type 2 Diabetes Mellitus (T2DM) patients with DFCs. Comprehensive foot examinations in the subdivision are conducted at the diabetic foot clinic by the trained foot care nurses. The study included HCWs based at the SDH with a minimum work experience in public health of at least six months. Intern doctors and nurses, and medical students were excluded from the study, as well as reluctant participants who met the criteria.

Purposive sampling was utilized to include HCWs from different work bubbles in each of the different work areas namely the Emergency Department, Outpatients, Foot Clinic, and the General Wards. A total of 20 HCWs were recruited into four FGDs with an average of five participants per FGD as data saturation was achieved and there was no new information obtained through the discussions [28]. Two of the FGDs were mixed groups which included a combination of doctors and nurses, and the other two FGDs included doctors and nurses in separate groups. HCWs were grouped according to their work bubbles as a result of COVID-19 work restrictions, with special consideration to ensuring power balance among participants and uniform gender distribution of doctors in particular.

### Data collection tool

A FGD guide was used to guide the interviews among the HCWs. The questions developed were based on the literature reviews which were in line with the research questions of this study. It had two sections including demographic questions of all the participants (like the number of participants, the age range of all participants, duration range of work experience of all participants and the gender distribution) and eight open ended questions. The FGD guide questions were tested before it was implemented for the FGDs, to allow the researcher a true time sense of conducting FGDs as well as adaptation of the questions used in FGDs. Three HCWs and experts were asked to review the FGD guide for its comprehensibility (by HCWs), and its ability to meet the aim of the study. The questions for the FGD guide was conducted in English.

### Data collection procedure

Ethical approval was obtained prior to commencement of the study. With COVID-19 work restrictions during the time of the study, flyers were advertised 2 weeks prior to commencing the study. Prior to commencement of FGDs, a pilot test was conducted on a group of HCWs to test the FGD guide questions and its relevance in capturing the research question. HCWs were grouped according to their work bubbles. Each participant was contacted a day prior to the scheduled Zoom sessions by the main researcher. They were thoroughly explained the purpose and aim of the study. They were reassured on the voluntary nature of the study which allowed them to exit the FGDs whenever they wished. Participants were informed of confidentiality of the study findings and the required audio recordings for the sole purpose of data analysis. All participants who agreed to participate was sent a soft copy of the participant information sheet. Verbal consent was secured prior to conducting FGDs via Zoom, and written consent was obtained in person by the main researcher. Each FGD lasted 45 to 50 minutes and all discussions were audio recorded. Notes were made during and after the FGDs which were compared with audio transcriptions for accuracy of data analysis.

### Data management and analysis

Audio recordings were obtained for all FGDs which were manually transcribed by the main researcher on the day of data collection. A review of transcriptions was done to correct errors and removed references of names and places ensuring anonymity for the participants. Once the transcriptions were clarified, data analysis was carried out. Thematic analysis was conducted in the following steps: first, the transcripts were read and revised for familiarization of the data, which was then organized in a meaningful and systematic way. We started marking preliminary ideas for codes that can describe the content. Then, we manually coded the data using different numbers. Transcripts were read line by line and similar phrases and words were classified using the number assigned to that word or concept. Themes were identified by grouping like phrases reflecting the perspectives of the participants, which were reviewed or modified to develop sub-themes. We used sticky notes along with white board to paste all the common codes in one color code which helped the researcher in identifying the themes clearly and grouping them. The themes after grouping were re-checked to see if they did not overlap or were allocated into the wrong themes. The final step was the write-up of research findings [29] with the presentation of themes and sub-themes supported by quotes from the transcriptions [28].

### Study rigor

To achieve study rigor the following four criteria was used: credibility, transferability, dependability, and confirmability [30, 31]. Credibility is the trustworthiness of research. In this study HCWs were contacted individually to confirm their availability and voluntary agreement to participate in FGDs which lasted 45 to 50 minutes. FGDs were audio recorded and transcribed on the same of by the researcher and were checked by the other researcher members. Peer debriefing was used to review and assess transcripts, methodology, and findings of the study. Triangulation of data source was done by using FGDs and note taking. Power imbalance was taken into consideration to avoid data bias, so participants were grouped accordingly to encourage open and free dialogue within FGDs. Transferability is the ability of the reader to apply and compare the study findings to their own settings. A thorough description of the participants and the research process has been documented in methodology. Dependability allows for replication and verification of the research process which was thoroughly documented in this study. A relevant study design was chosen to conduct this study. The developed questionnaire was tested in a pilot study. The interviewer knew the local language and used simple communication strategies to facilitate collecting data. There was a systematic approach to identify the codes, sub-themes and themes. Transcriptions were reviewed to check for accuracy of documented FGDs. Confirmability is achieved through transparency of data collection, analysis and interpretation of the research process. The process of conducting this research was checked by an audit trial. The data collection, transcribing and analysis were checked by the co-researchers. The quotes from the study participants were used to generate themes. The place and dates of data collection was chosen based on an agreement between researchers and study participants.

### Ethical consideration

Ethical approval was obtained from the College Health Research Ethics Committee (CHREC) of Fiji National University (FNU) with the ID#053.21 and facility approval from the Sub-Divisional Medical Officer (SDMO) for Nadroga-Navosa. All willing participants were thoroughly informed of the study purpose and aim, participant confidentiality, and the voluntary role of participants. All voluntary participants and were provided with participant information sheets. The soft copy of the documents has been saved in a flash drive and has been locked with the consent forms in a secure place. The transcribed FGD and recordings has also been kept in a flash drive, locked securely for at least 3 years after which it shall be destroyed. Both verbal and written consent was secured prior to conducting the online FGDs.

## Results

### Participant characteristics

As Table 1 shows, there were 20 participants, the majority of whom were nurses (60%), with even distribution among the two major ethnic groups. There were more female (80%) to male participants. The majority of the participants had a work experience of 1–5 years (35%) and over 15 years in public health (35%).

**Table 1:** Participant general characteristics (n = 20).

Characteristics	Categories	Frequency	Percentage
Gender	Male	4	20
	Female	16	80
Ethnicity	I-Taukei	10	50
	FID*	10	50
HCWs	Medical Officers	8	40
	Nurses	12	60
Years of work experience	1–5 years	7	35
	6–10 years	4	20
	11–15 years	2	10
	Over 15 years	7	35

* Fijian of Indian Descent

### Themes and sub-themes identified

The thematic analysis of data resulted in four major themes which are: HCWs’ perceptions and practice of foot care; factors affecting foot care; creating awareness; and strengthening foot care practices. From these themes emerged sub-themes and categories summarized in Table 2.

**Table 2:** Themes and sub-themes of HCW FGDs.

Theme	Sub-theme
HCWs’ perceptions and practice of foot care	Comprehensive foot care Advising patients Barriers of foot care practices
Factors affecting foot care	Patient factors Resources Healthcare services
Creating awareness	Patient education Training HCWs Improving health care services
Strengthening foot care practices	Continuity of patient care Boosting healthcare services Policy makers

Each participant is coded with a number (1–20) as well as the FGD (FGD 1–4) to which they were included (noted in the quotation references). The quotation references presented are indicated at the end with the participant quoted and FGD from which the responses were derived. The abbreviations used after the perception quotes for the HCWs are RN (for Registered Nurse) and MO (for Medical Officer).

### Theme 1: HCWs’ perceptions and practice of foot care

The first theme explored HCWs’ knowledge of what comprehensive foot care entails, the type of advice relayed to patients, and the different aspects of foot care barriers.

#### Comprehensive foot care

Respondents perceived foot care holistically and integrated their knowledge of diabetes complications into their approach of comprehensive foot care and patient advice.

*“For comprehensive (foot care) we go to all the other risk factors they have like hypertension (HTN). If they have HTN, poor control of sugars–we optimize their sugars, if they are smoking–we advise on smoking cessation …the risk factors we advise and manage that accordingly. That could be part of our comprehensive foot care.”* (FG4, a MO)

They were able to verify that an ‘at-risk foot’ is beyond managing DFUs. Examining patients for tell-tale signs of neurovascular changes due to diabetes as well as routine toenail care are important aspects of diabetic foot care.

*“It’s important to check for the colour of the skin.”* (FG3, a MO)

*“Reflexes and sensation (of feet)*. *Sensation*, *very important*, *to see if the patient can feel*. *And on the same note reflexes*.*”* (FG2, a RN)

*“With all of those*, *you can also check for pulsations (of feet)*. *You can feel for pulsations if they’re weak or if there’s any difference on bilateral inspection (of feet)*.*”* (FG2, a MO)

*“When they go out into their own areas*, *those are the things they should be teaching the patients–when*, *how often*, *how to cut their toenails*. *Those are the things that are very important*.*”* (FG1, a RN)

There were varying opinions on the frequency of foot screening for diabetic patients which may translate to clinical practice depending on the attending physician. Opportunistic foot checks either at home by patients and family, or during routine hospital visits were positive ways to encourage foot screening.

*“I would say, if I am given a chance to make my own perceptions. So, for those (doing) home care (foot checks) every day. So, for hospital care (foot checks during) every diabetic clinics. And diabetic clinics would be according to their PEN assessment. So that’s my take on that.”* (FG3, a MO)

Respondents had different opinions on current foot care practices in the Nadroga-Navosa subdivision based on personal experience. Despite adequate diabetic foot care knowledge of HCWs, there was a marked difference in practice at SDH in comparison with a peripheral health centre. SDH foot care involved managing DFUs and DFS cases, while foot care at the peripheral health centre entailed screening for DFCs. Barriers of foot care practice are explored later in this chapter.

*“I think for her (foot clinic nurse–SDH) it’s just the DFS cases. It’s not until she’s told to take a look at the foot and then she goes. But I have seen sometimes our Mos (medical officers) too they tend to check.”* (FG4, a MO)

*“When I was at the health centre…We had a diabetic foot nurse*, *the clinical nurse*. *She used to do random checks on patients regardless of like*, *if the patient had ulcer or any sepsis at that stage … But in our current SOPD (at SDH) we just do checks*, *on the foot sepsis now*. *Neuropathy and other things I’m really not sure at this stage*, *(if) they’re still checking or not*.*”* (FG4, a MO)

#### Advising patients

HCWs’ approach to patients during educational talks and clinics impact how foot care advice is received by patients.

*“The thoroughness of the advice, how you approach the patient, how you evoke a positive change those things are very important… that can have a positive change in how they can look after their foot.”* (FG3, MO)

Specific strategies or approaches suggested by the participants. For example, motivational interview can be utilized as a tool to evoke change among patients; with more effort towards patients with poor compliance to diabetes management and foot self-care.

*“So, I’m like just going to touch again on my favourite thing, motivational interview…So health information is not always equal to behavior change…Most of the patients are already aware that (if) they don’t control their sugar, they will end up with an amputation…But then on the part of the physician…it’s very, very important to dwell on those who have poor compliance.”* (FG3, a MO)

All respondents were well-versed on diabetic foot care offering foot care advice pertaining to foot self-care and hygiene, proper footwear, and avoiding harmful foot care practices.

*“For diabetics… it’s (like) the way you look after your face in the morning, you look after your foot like that. You tell them (patients) ‘ok you take care of calluses, you nip your toenails, you put oil there after washing with soap and water and drying in between the toes, and you wear well fitted shoes, don’t walk around barefoot.”* (FG3, a MO)

*“Proper sizing of shoes*, *is one of the advice I’ve given… not too tight*, *not very closed*. *Clean your feet*, *wipe your feet and examine your feet*.*”* (FG3, a MO)

*“And sometimes when they have those leg pains*, *they try to put their leg into a basin of hot water to keep their legs warm…they come up (to SDH)…and they have blisters…which really makes them have those DFS…”* (FG4, a RN)

Due to patients’ perceived barriers, the specific roles envisioned for family members. It was encouraging to note that respondents also considered it important to involve family members in foot care practices at home.

*“If the patient is not able to see the feet by themselves, they can ask one of the family member to inspect the foot for them.”* (FG2, a RN)

Aside from routine foot care education and practice, patients need to be encouraged on adequate diabetes control to prevent diabetic complications like peripheral diabetic neuropathy amongst other systemic effects.

*“Also to ensure that they are taking medications on time, their diet control comes with it, and being compliant with their medications, their clinics…If they are seen, they need to comply with the (diabetes) medications we give them, (and) their review dates.”* (FG3, a MO)

There were perceived reasons for delayed presentations, potential barriers faced by patients, and suggestions from the participants on how to overcome these barriers. Early hospital presentation is essential for the effective treatment of simple wounds that have the potential to progress to DFUs.

*“We also advise our patients as soon as they find an injury to their foot for them to present to hospital and I think the main thing about that is so that we can see the severity of that injury so we can start them on antibiotics if they need it.”* (FG 4, a MO)

This can be enforced among patients through adequate and regular patient education and awareness of diabetic foot complications and its consequences.

*“I think creating more awareness on foot related diseases and how serious infection can go leading them to amputation…Like we see a lot of very bad cases, gangrenous foot…So, we have to create awareness and tell them…They can lose their limb, (be) unable to walk and further complications due to their amputation can occur.”* (FG3, a MO)

Due to need for social supports, foot care should not be limited to patients alone. Family members should be involved for continued care at home.

*“We also advise our patients if we get an opportunity for somebody younger who is more literate in the family to come (for clinics) and we talk to them. Sometimes patients are in denial, other person who is looking after the patient, he/she has the good knowledge, they’ll bring patient to hospital on time and monitoring is done at home.”* (FG2, a RN)

#### Barriers of foot care practices

Without adequate knowledge, HCWs will not be able to confidently manage and practice routine diabetic foot care checks for patients.

*“Sometimes when we have cases in Women’s ward and Men’s ward, we have to refer the cases to her (foot clinic nurse)…And to improve on that we can like, you know, have some other nurses to like go for trainings…in the wards there’s no foot nurse trained.”* (FG1, a RN)

Busy SOPD clinics reduces the quality of patient care provided by HCWs due to limited consultation times.

*“I know we see more than 50 patients in SOPD so we cannot be going through and checking everyone’s foot basically.”* (FG4, a MO)

Lack of basic medical resources for foot care largely impacts low-income patients who cannot purchase their medical supplies.

*“Another problem is the ‘out of stock’ situation… Sometimes the only patients who get better wound treatment is the ones who can afford to go and buy the ointment and the gauze. Even sometimes the gauze is out of stock. So, when supplies are out of stock that’s also a big hinder.”* (FG1, a RN)

One respondent promotes the use of herbal medicine for treating diabetes and DFUs, based on her personal experience. The negative implication of this is that patients will opt for herbal treatment as a first-line of care, further delaying presentation and initiation of appropriate medical treatment. This will affect their treatment progress and bring more cost for the patients and health system. It also increases the potential risks associated with such practices.

*“We have come up with herbal medicines that can cure–reverse diabetes, like mango leaves. Even with the healing of the wounds they are using ginger and honey, I always encourage them to use it. Just advise them to use it if they know that it can heal. But it heals. I’ve come across cases that I’ve looked after.”* (FG1, a RN)

### Theme 2: Factors affecting foot care

#### Patient factors

A multitude of factors posing as foot care barriers were identified by all respondents. Commonly mentioned barriers were the lack of patient knowledge on diabetic foot care, language barrier, and low level of education.

*“Lack of educational awareness and not enough educational materials that we need to be providing for them at the hospital. Maybe we should get more pamphlets or more of our staff standing while they are waiting in the waiting area to actually teach and talk to them about basic foot care in a language that they understand.”* (FG3, a MO)

Herbal medicine is largely influenced by culture, which is a prominent and existing practice in Fiji that poses a great challenge for HCWs when dealing with DFCs. The use of herbal medicine as a first resort to addressing any diabetic foot problem delays patient presentation to health care facilities. Late presenters are often admitted with severe DFS requiring urgent medical attention and amputation.

*“And we usually see most of our diabetic patients they go straight to herbal medication…and they wait. So during the clinic we usually advise them as soon as you find an ulcer or even a blister please get a medical opinion as soon as possible. Don’t wait for a week or two and go around and put herbal and all that as well.”* (FG 4, a MO)

The Nadroga-Navosa subdivision spans across vast terrains, comprising of a large part of coastal area that extends up to the mountainous regions of Navosa. Geographical challenges have always been a major hindrance for patients and HCWs alike due to the availability and affordability of transportation to seek medical attention.

*“Geographical factor is a main one (barrier to foot care). If they leave very far away, then they know they have infected skin and they are unable to come. Even with the doctors’ advice…to come for review…they are not able to make it, (and) it worsens their condition (diabetic foot infection).”* (FG3, a MO)

Financial constraints affect patients’ diabetes and foot care management.

*“Economical status. Some of them they afford to wear shoes, some don’t have proper shoes.”* (FG1, a RN)

*“Other factors could be controlling their CBG and availability of the medications at our hospital pharmacies*. *And patients not being able to afford CBG control medications from private pharmacies*.*”* (FG3, a MO)

Social support is essential for all patients. Family members should be involved in patient education and care. Factors associated with aging and diabetes like poor eyesight and reduced mobility may prove challenging for patients to do routine foot checks at home.

*“And then there should be also a family member that’s to be with the patient so that we can also relay the message to the family member to be aware of that. Sometimes the patient forgets then the wife or that family member will be able to uh inform the patient again that he has to look after his foot.”* (FG1, a RN)

Patient health seeking behavior impacts disease management and outcome of DFUs. Early presenters can receive timely treatment with improved DFU outcomes.

*“The health seeking behavior of the patient plays a very important role, like for our settings. Some have good health seeking behavior which can be an enabler. They come to a facility as soon as thy notice something, even a slight discoloration.”* (FG3, a MO)

However, a patients’ health seeking behavior may be influenced by their knowledge of diabetic foot care and complications; their personal beliefs and preference for traditional medicine to medical treatment; geographical location; financial stability; and family support. The added fear of contracting the COVID-19 infection has led patients to endure their DFUs at home instead of seeking medical attention.

*“So, what we are seeing now is a lot of very badly infected diabetic foot coming to hospital. So, a lot of them might just be afraid of getting COVID in the hospital,”* (FG2, a MO)

#### Resources

Inadequate staffing is a chronic issue, which negatively impacts foot care practices among HCWs.

*“Staffing. Sometimes when we are short staffed we don’t have all the time to check (routine foot checks).”* (FG1, a RN)

The lack of medical supplies is another ongoing concern which affects productivity as well as quality of care.

*“We need resources as well. There was a timely lately when it was out of stock from FPBS (Fiji Pharmaceutical & Biomedical Services), patient comes in for clinic but we are not able to debride. We’ll go around looking for it for half an hour, one hour. If we manage to get one then we do it.”* (FG2, a RN)

Time constraints among HCWs due to patient load is a limiting factor to proper foot checks by busy nurses who have to manage different work areas. The diabetic foot nurse sees her diabetic foot patients during their routine Monday clinics, but also has to attend to outpatient cases who come in for daily dressings and injections (repeat treatment).

*“Like for me, with my experience as a foot care nurse. If I do a good foot care it takes me 10 to 15 minutes up to 30 minutes sometimes. So if I start doing a foot care on a busy Monday, my all repeat treatment are held.”* (FG2, a RN)

All SOPD clinic rooms at SDH were converted to isolation rooms to cater for COVID positive cases requiring admissions. The unfavorable set up of diabetic foot clinics was reflected in the poor quality of care afforded to patients during their hospital visits.

*“Our work place, we don’t have enough space (foot care room merged with outpatient treatment room)…if we have a foot care room somewhere and another person continues treatment. So what we are basically doing is we do whatever we can in that short period of time. And not sufficient care and debridement or cleaning up is done or patient education.”* (FG, a RN)

#### Healthcare services

Disruption of patient care after hospital discharge was a highlighted concern. This may be one of the factors for patients representing to hospital with severe DFUs after discharge. This can be addressed by ensuring continuity of patient care in the community through effective communication between the discharging team and the district nurses.

Many of the respondents discussed the negative implications of COVID-19 on the healthcare services in Fiji. Reorientation of healthcare services and resources towards the crusade against COVID-19 took precedence, so routine NCD clinics and home visits were disrupted.

Impact of COVID at peripheral health facilities within the medical area:

*“And at the moment a lot of our staff are engaged in other activities. Our health centre staff mostly are involved with (COVID) vaccination and home-based care…So, the health centre nurse who usually looked after the medical area, usually did home visits for a lot of these diabetic patients–take care of their wound, give their medications. And now what happened, she was in isolation (due to COVID) and there was no one caring for them. Only one nurse would go relieve for MCH only for one week but that isn’t enough for these patients’ requirements and home care.”* (FG2, a MO)

Impact of COVID at SDH:

*“So, our current COVID situation—we are not doing any foot clinic, we are not doing any SOPD checks…So we are sitting on a big time-bomb of NCD crisis when this COVID is over. We’ll be battling with diabetic foot complications and other diabetic complications.”* (FG3, a MO)

Poor patient follow-up during COVID-19 influenced the increased threshold for diabetic foot admissions at SDH. Early intervention of diabetic foot infections through hospital admission was aimed at reducing the progression to DFS and lower limb amputations. Unfortunately, timely surgical intervention for diabetic foot infections could not be achieved for all patients due to strict COVID-19 protocols at the time. This was even more challenging for SDH as all COVID nasopharyngeal swabs (NPS) had to be transported to neighboring health facilities for processing.

*“The other thing is how much we can accommodate in our (healthcare) system at the moment. Like right now even patients going across for surgeries…gets delayed by a few days. Why? Because we need to get their swabs (nasopharyngeal COVID swabs) arranged, make sure they are either negative or positive…And this kind of delays the process as well.”* (FG2, a MO)

### Theme 3: Creating awareness

#### Patient education

Incorporating NCD and diabetic foot care education and awareness in school may be a way to reinforce public awareness and education.

*“Maybe foot care should be taught at a young age too like when they are still in school…Maybe if they get the habit of being taught when they are young, when they are old they still know.”* (FG1, a RN)

Routine and opportunistic foot screening is always an opportunity for patient education and awareness.

*“We can always ask the foot care nurse to always have foot care clinics every time they come in for SOPD.”* (FG4, a MO)

Refreshing patient knowledge on diabetes and foot care practices during every clinic visit reinforces patient education and awareness.

*“I think one of the factors could be our SOPD clinics and if we do focus on the DM clinics and every week we do have our physiotherapist and our foot clinic nurse and our dietician advising on all this, the repetition might help people remember points to take good care.”* (FG3, a MO)

The community is immersed in mass media which is a great platform to reach a large audience in the comfort and confines of their home, especially during curfew and movement restrictions due to COVID-19.

*“Increasing awareness on our radio stations, our feedback stations and our TV of how people can look after their feet. And also to present early when they have complications. Especially during this time, during this COVID situation. Most of the time we are just at home, on home isolation, after curfew. So maybe the government can use media now to raise awareness on NCD issues especially like for DFS.”* (FG3, a MO)

Providing verbal as well as written forms of educational advice on diabetes and foot care in English and their vernacular gives patients the opportunity to reflect on the advice given when they are at home.

*“Maybe we can have those pamphlets that patients can take with them upon discharges. For their additional information. We can have them in all languages so upon discharge they can take it with them for better understanding.”* (FG4, a RN)

Foot care workshops and trainings tailored to create interactive patient education and awareness on diabetic foot care.

*“We can also include our patients…In that the patients will be motivated. They will be hands-on on…diabetic foot care. Why can’t we do a workshop for them, get them involved as well.”* (FG4, a MO)

Improving patient accessibility to healthcare services through routine outreach clinics allows for foot care screening, education and awareness.

*“I think we should do more outreaches. Like mainly people don’t present to us because of their geographical and financial difficulties. We should increase our outreaches to the interior communities where they are unable to present to us.”* (FG3, a MO)

#### Training HCWs

Foot care trained staff can conduct in-house teaching sessions for hospital staff. This can help build confidence among other HCWs to encourage the practice of foot care in hospital.

*“If they can conduct in-house training for diabetic foot sepsis. We have nurses who have attended workshops and if they can hold sessions for the hospital for us to be well informed. So that when we come across cases instead of calling for the foot care nurse we are there to assist. We can reduce our amputees.”* (FG4, a RN)

Empowering HCWs and community health workers by providing training opportunities on diabetes foot care will help decentralize services to the peripheral health centres. This will ease the patient load on diabetic foot nurses at SDH and benefit patients in the community.

*“If everyone gets through that foot care nursing…If we go through the workshop, like at least we’ll have a brief idea what all we can manage in the wards instead of taking the patients to the foot care nurse…the public health nurses should be involved with this foot care. Because they are the ones who go to their areas which they’re assigned for and all the zones…”* (FG4, a RN)

Community health workers or village health workers are a great asset to public health teams because they are our advocates in the community. Involving them in community foot care trainings and screening will help HCWs identify at risk cases in the community who might otherwise be overlooked.

*“Our subdivision we tend to use our village health workers as well and they’re trained in some ways which is very good and they go out of their way and they alert us on which patients need medical attention…So one good thing about it is that we’re using them as well, our village health workers, to follow up on patients especially when they are staying far away.”* (FG4, a MO)

Foot care training should not be limited to HCWs alone. Involving community leaders are a great way to promote awareness in our communities. Having influential community members involved in diabetic foot care may encourage early health seeking behavior amongst community members.

*“The other thing we can also encourage is more education in our villages and in our communities through training more of the village health workers. Also training of community leaders you know, youth leaders on DFS and its care and its complications.”* (FG3, a MO)

#### Improving healthcare services

Mobile foot clinics can be initiated as a way to ensure high risk diabetic patients who have trouble attending foot clinics at SDH are adequately followed up.

*“I think it would help if we had the list. Our foot care nurses, the patients that they see. Because to my understanding they have appointments. So, if they had a list of names, the location and all, it would make things easier too for us to follow up.”* (FG4, a MO)

Conducting a week of foot care awareness at SDH and having specialist consultation and care available for patients is another way to boost foot care services.

*“We should have like a foot clinic for one whole week for everyone who has foot problems they can come and see her (foot clinic nurse) and the specialists. That way we can create more awareness…Maybe we should have pamphlets too…if we don’t have time to explain we can make them read it at home.”* (FG1, a RN)

Utilizing SOPD clinics as a platform for diabetes and foot care awareness through educational talks and demonstrations.

*“You know how we have SOPD, while they are waiting around to be seen they can be given pamphlets, they can be given educational materials to help them know the risk factors and be able to know actually when to present*

*…the signs of DFS*. *One of the things that is also important is to teach them how to actually look after their feet in a very basic (way) and in a way they actually understand*.*”* (FG3, a MO)

### Theme 4: Strengthening foot care practices

Respondents shared their views on clinical practices which can be strengthened and improved to bolster foot care practices. The sub-themes identified through discussions were continuity of patient care, boosting healthcare services, and the role of policy makers in foot care.

#### Continuity of patient care

Opportunistic screening for diabetic foot complications and routine foot care for all admitted patients is a way to capture new cases. All discharge summaries should include diabetic foot screening summaries which are provided for patients and HCWs for further follow-up at their respective clinics.

*“We have it (diabetes foot screening form) but we are not widely using it. We should have the check list in our admission folders where the admitting nurse does all the checklist of the diabetic foot care for the patient…the progress while admitted in the ward…the foot assessment upon discharge. And that form comes back to the clinic. Wherever patient attends clinic and then we continue to see. In other words that measures the care that we deliver to our patients in order to save that diabetic foot.”* (FG2, a RN)

Oversight of HCWs on timely and effective foot care practices must be avoided to save a limb and a life.

*“For health workers too to recognize the problem and refer the problem. Most of the time we are busy with other problems we forget about the small things that could become worse like small wounds that could become worse.”* (FG1, a RN)

Routine domiciliary visits by the respective health centre zone nurses can help identify high risk cases in the community that need special attention and early referrals for further treatment.

*“Most of our cases we see them come out from the wards and they end up coming back again. So I think there’s a weakness in the domiciliary visits. I think they need to strengthen that to check on these patients especially the ones getting discharged. Because it’s no use you going home with a small FFA and you come back with an ulcer, BKA or AKA.”* (FG1, a RN)

Follow-up care in the community hospital discharge is crucial in ensuring full recovery of patients. This highlights the advantage of frequent diabetic foot reviews and patient education towards prevention of DFCs and DFUs.

*“I believe that our foot care nurses and us as HCWs, we have a very important role in helping somebody get through DFS. My mum, she’s diabetic and she had suffered a burn to her first toe. She did not know she had burned her toe because of the neuropathy… she was referred on time and she had her surgical debridement on time. And not only that, the aftercare, it was very, very important for her. …it had healed through her going to her own nearest HC where she was looked after by a dedicated foot care nurse …-”* (FG3, a MO)

#### Boosting healthcare services

Holistic patient care goes beyond addressing their medical issues. Assisting patients by getting them enrolled into government social welfare benefit schemes improves patient accessibility to the healthcare services they need.

*“When we are looking at our patients, especially area medical officers, to have a more holistic approach for patient care…apart from looking at that limb or apart from just looking at that patient, we see the challenges that they are facing…So if they are coming from very far away and they are having issues with transportation because of financial issues.”* (FG3, a MO)

Timely intervention of diabetic foot injuries that have the potential to progress to DFS and lower limb amputations can be met by having a low threshold for hospital admission and treatment.

*“Maybe when they do present to us with a little sore or something that’s pointing towards like it might end up with a DFS or an amputation. Considering their financial status, their background, their geographical location. Maybe have a low threshold for admission or get a second opinion always before you send them away or they end up losing their limbs.”* (FG3, a MO)

Despite the temporary halt in routine clinics at the health facilities during the COVID-19 pandemic, the field teams (SORT) involved in contact tracing for COVID cases were able to identify and refer DFS cases in the community. This reiterates the importance of outreach programs and domiciliary visits to the community.

*“We have our SORT team. Like during this COVD situation we have this home-based monitoring. And there are some cases of diabetic foot sepsis we managed to pick up during this time. So one good thing is our team present directly to the patient themselves if they have some difficulty presenting to hospital or anywhere for that matter or to the health centre.”* (FG4, a MO)

Empowering our communities with knowledge through awareness and education programs that address NCDs, diabetic foot complications, and foot care practices gives the community ownership of their health and well-being.

*“The thing that I remember that used to be done (at the health centre)…the foot care services from CWM had started to organize…peer educator groups…in these groups there used to be a health worker, with either a community leader, a community youth group or a VHW. They would identify their own diabetic patients or NCD patients…these groups they would be involved in foot check, CBG check and BP check…they would … be counseled on how to look after their feet and to present early for danger signs”*(FG3, a MO)

#### Policy makers

Reintroducing foot care into current ministry operational plans is a way forward in re-prioritizing foot care.

*“From (the) last two years that indicator has been removed from the operational plan…but I felt that was a very important indicator. And so if we are able to reverse a foot complication by having a patient attend a foot clinic, de-sloughing and looking after it well, it’s a big achievement. And even if we are able to save 10 feet a year that’s 10 lives, 10 families that we are making a difference for.”* (FG3, a MO)

Engaging community health workers in health promotion and giving them ownership of advocating for NCDs, namely diabetes and foot care can be utilized as a way to reach out to the community.

*“I feel community health workers are our very important eyes and ears on the ground and they are our mouthpiece, our point of advocacy in the community. Having them trained is one aspect in making sure this is followed, but making sure that they carry on with that promotion, that awareness. This can be reflected in the monthly report that they submit.”* (FG3, a MO)

Conducting audits on all healthcare facilities in the Nadroga-Navosa subdivision can be utilized as a steppingstone to strengthening foot care clinics and current practices in place.

*“So, like we have 15 health facilities in Nadroga-Navosa.…I would say most, if not all our health facilities have foot care trained nurses…how well they are able to function and reverse a foot complication is another debate…so having someone audit these practices is another key step that needs to be undertaken.”* (FG3, a MO)

## Discussion

This study was aimed to explore HCWs’ perspectives on diabetic foot complications and challenges of foot care management in Fiji. Comprehensive foot care was perceived as holistic care that involved foot examinations as well as addressing important risk factors like smoking, and co-existing comorbidities like hypertension. Respondents discussed key areas of foot examination namely inspection, neurological assessment and vascular assessment in line with clinical recommendations [32–34]. But toenail care and patient teaching on this was often overlooked as part of routine foot care. Recent practices have shifted the focus of diabetic foot examinations to DM patients with acute or active ulcers. During the study, the main barrier was the nationwide COVID-19 health restrictions that temporarily suspended all routine clinics.

This study showed different approaches and strategies that were suggested by the patients. Patient approach and motivational interview was highlighted by respondents as an essential way of establishing rapport and evoking behavior change by exploring and resolving ambivalence to change [35, 36]. However, its impact on footwear practices only shown some short-term positive effects which may require additional or adjunctive therapy in the long run [35, 36].

Patient foot care advice provided by respondents adequately covered all aspects of foot care that are in line with literature [32–34]. An active foot care advocate promoted the use of herbal medicine to cure diabetes and foot lesions. This was based on her experience with previously successful patient treatments using herbal medicine, though further research is required to endorse the safety and success of such practices. The danger lies in the potential toxicity of herbal medicine, let alone the combined effect of mixing both forms of treatment [37, 38].

Barriers to foot care practice is an ongoing and universal issue. Some barriers identified by respondents include: the lack of knowledge of HCWs to confidently apply foot care in their daily work; busy SOPD clinics resulting in patients being overlooked for routine foot checks; and the lack of resources such as medications, dressing materials, and staffing. These findings are also underpinned in previous literature [39–42]. And the suspension of routine SOPD and foot clinics due to the COVID-19 pandemic has greatly affected foot care practices [43].

Most of the patient factors identified by respondents were barriers to foot care which include: lack of foot care knowledge; low level of education and language barrier; financial constraints that limit patients from purchasing proper footwear, purchasing of medications and fare to travel to hospital for clinics or reviews; and geographical location as a barrier due to limited accessibility to health services with costly means of transportation [9, 12, 41, 42, 44–47].

Social factors can prove to be barriers or enablers [45]. Patients with improved family support and large social networks (community involvement with neighbors and friends) have been found to have better foot self-care practices [48]; and a lack of it is a barrier to foot care practice [49]. But communal activites like sharing a family meal makes it difficult for individuals to adhere to DM dietary recommendations [45]. System factor barriers which was identified by respondents included the suspension routine SOPD and foot clinics due to national COVID-19 restrictions [43]. This underpins the need for efficient planning by stakeholders for effective healthcare pathways to allow efficient service delivery for all patients now and in the future. The alteration of health services highlights the need to promote and strengthen DSME for all diabetics [43].

Availability or lack of resources are a very large determinant of foot care practice. Some foot care barriers identified by respondents include inadequate staff, lack of essential medical supplies for DM and foot care, time constraints and limited clinic space for proper foot clinics. Inadequate staffing with only few specially trained foot care nurses is a barrier [9, 40, 42, 50]. Foot care delivery becomes strained among the foot care trained HCWs [39, 51]. Lack of medical supplies such as essential medications, dressing materials and other consumables is a challenge for patients and HCWs alike at SDH [40, 45]. Time constraints, overbooked clinics and lack of trained staff result in long queues and overburdened HCWs [23, 45, 50]. The lack of stuctural space for proper foot care has been due to the reallocation of staff and resources to accommodate for the care of COVID-19 cases at SDH. The end result was delayed intervention for DFS cases [46].

The proposed awareness strategies by respondents were targeted at the three main groups that are the main influencers of foot care and its outcomes: patients, HCWs and the healthcare system. Patient education is one of the main discussion points in this section and is supported by literature as one of the ways of imporoving foot care practice [12, 44, 46–48]. Diabetes Self Management Education (DSME) has been underpinned by Bonner, et al., (2016) as an essential way of preventing diabetes related complications, like foot problems because patients who were not offered DSME were at a four-fold increaesd risk of developing diabetes related complications, like foot problems. Non-expensive education strategies found by Negarandeh, et al., (2013) and Sari, et al., (2020) to be beneficial for low health literacy are teach back and pictorial image education strategies which were used to improve DM education and medication and diet adherence. This form of education strategy can be tailored for education and awareness of DFCs and foot care among DM patients [52, 53]. Group care has been found to be more effective in the long term care of diabetes, in comparison to usual one-to-one consultations which is more ideal for acute, short term treatment [54, 55] because patients are amongst peers and their shared experiences of managing DM gives them a more positive outlook on DM care, teaches them problem solving skills that helps them adapt to daily care, and is a form of social support for patients [9, 42].

Educating HCWs is essential because they are the main source of patient education, awareness and information. Having adequately trained staff will help improve the delivery of foot care, HCWs will be more confident in carrying out foot care practices at work, and they their enthusiasm for patient education and awareness will be improved [9, 42]. Motivational interviewing skills was also porposed by a respondent as an essential tool for education, awareness and behaviour change [35]; which should be incorporated into foot care trainings among HCWs. Diseminating foot care information through training of community health workers has been proposed as an effective way of educating far reaching communities with limited access to mianstream health services. McDermott, et al., (2015) and Kane, et al., (2016) have found improvement in patient DM care with trained community health workers assisting in patient DM care in remote areas, supported by a clinical outreach team [56, 57]. However, Jiang, et al., (2021) found no significance in the self-efficacy of foot care during a 2-year follow-up of type 2 diabetics on community based self-management intervention, and suggests future efforts focused on strengthening foot care among patients [55].

Accessibility of foot care services is a challenge for remote communities, which can be overcome through the provision of mobile foot clinics. Utilizing Diabetes Day for annual DM and foot care awareness can help improve the perceived importance of DFCs and foot care [9]. Patient access to a DM educator, an ophthalmologist or even the dietician has been found to patient knowledge [58] which can be achieved during routine clinics. Patient behaviour is found to be positively influenced by education in the short term but there is limited evidence that patient education prevents the primary outcome as adequate knowledge does not mean actual foot self-care behaviour [59]. This is supported by a study conducted by Yuncken, et al. (2018) that highlighted that many diaebtic patients who received diabetes and foot care education during podiatry consultations had poor education recall over the 6-month period. Education recall can be affected due to many factors like low literacy levels, mild or moderate cognitive impairment and competing priorities of the patient [60]. Dorresteijn, et al., (2014) underpins the importance of continuous patient and caregiver education on DFCs and care [59].

Continuity of patient care is essential to maintain after patient discharge to their respective medical areas. Some respondents suggested routine foot screening with every diabetic admission, and referred care to the reviewing HCWs; a form of opportunistic screening [9]. This creates an opportunity to capture patients who are overlooked during routine annual diabetic foot screening checks which are essential for identifying at risk patients with peripheral neuoropathy and peripheral artery disease [13]. HCW awareness and education will allow timely patient referrals and treatment highlighted by Pankhurst & Edmonds (2018), and Littman, et al., (2021) [40, 46]. Holistic DM patient care also includes addressing psychological aspects of patient [45]. System level barriers such as early admissions of DFS can be offered to reduce amputations [45]. The lack of telecommunication services to some rural areas in the Nadroga-Navosa subdivision made teleconsultations impossible for individuals homebound by COVID. Similar challenges were highlihgted by Gupta et al., [43]. Alternative health services were activated with HCWs doing home consultations and case retrievals when required.

Policy makers have the potential to redirect services to prioritize foot care. This can be achieved in several ways, by incorporating foot care as part of ministerial goals. Extending foot care responsibility to involve community health workers [56, 57] with recommendations on focussing group activities to strengthen foot care [55]. Assessing current foot care practices through routine audit [9]. Foot care SOPD audits can be incorporated into routine PEN Model audits that are routinely done nation wide. Each of the PEN folders that are audited can also be assessed for diabetic foot classifications.

### Strengths and limitations

This is the first qualitative study in Fiji to explore the perspectives of HCWs on diabetic foot complications. The study reports conducted in high methodology quality as all the process was conducted rigorously. Some study limitations include the exclusion of HCWs based at the peripheral health facilities. Their response may have been helpful in identifying current foot care practices and challenges in the rural as they are the advocates in the community. FGDs was the only method of data collection and the study setting was limitd to the Sigatoka Subdivisional Hospital alone. The study findings may not be generalizable to tertiary healthcare facilities who have a more complex system of operation and challenges; but may be more relatbale to other subdivisional healthcare facilities managing diabetic foot complications at the grass roots.

## Conclusion

Through the FGDs, the HCWs easily identified barriers to foot care knowledge and practice that included patient, HCW, and healthcare sytem factors. Lack of patient knowledge, financial constraints, geographical location, personal beliefs, the fear of COVID, and cultural beliefs and practices namely herbal medicine; were commonly identified barriers for patients [50, 61, 62].

The HCWs demonstrated adequate foot care knowledge, with many expressing the need and interest for more foot care training opportunities among HCWs. Many foot care barriers were identified; namely the impact of COVID on healthcare services and resources. The most reiterated barrier was the lack of resources: inadequate staffing, lack of essential medications and medical supplies, inadequate space to conduct proper foot care, and the lack of time afforded to foot care checking and education. The recommendations made by HCWs was soley based on these barriers: improving foot care awareness, education and knowledge among HCWs to improve the perceived importance of foot care among other HCWs. System level barriers like recruiting more staff, ensuring essential supplies and medications required for foot care are available at all times, strengthening on home based care; which is when the HCWs go out into the community and see and management cases accordingly.

Some noteworthy practices which have been previously used such as incorporating diabetic foot prevention as an indicator for health ministerial operational plans, and mobile foot clinics involving HCWs and community members should be revived.

The analysis that is presented in this study may help convey valuable information for future research conducted on a larger scale that can help improve diabetic foot complications and amputations in Fiji. The data can also be used by stakeholders to invest in diabetic foot care education and early screening for foot complications through improved policies, infrastructure, and resources.
